# Supplementary material for: Prevalence and Impact of Cerebral Microbleeds on Clinical and Safety Outcomes in Acute Ischaemic Stroke Patients Receiving Reperfusion Therapy: A Systematic Review and Meta-Analysis
Source: Biomedicines. 2023 Oct 23;11(10):2865. doi: 10.3390/biomedicines11102865 (PMC10604359; doi:10.3390/biomedicines11102865)
Supplement: Supplementary file 1 [file biomedicines-11-02865-s001.zip › biomedicines-2661521-supplementary.pdf]

biomedicines

Supplemental Information

Prevalence And Impact of Cerebral Microbleeds on Clinical And Safety Outcomes In Acute Ischaemic Stroke Patients Receiving Reperfusion Therapy: A Systematic Review And Meta-Analysis

Shraddha Tipirneni, Peter Stanwell, Robert Weissert and Sonu M. M. Bhaskar *

* Correspondence: author: Dr Sonu M. M. Bhaskar, MD PhD FANA
Ph: +81 90 9274 1265
Email: Sonu.Bhaskar@globalhealthneurolab.org / Bhaskar.Sonu@ncvc.go.jp

List of Contents

S1. Search Strategy

S2. Supplemental Tables

Supplemental Table S1. Preferred Reporting Items for Systematic Reviews and Meta-analyses PRISMA (2020) Checklist

Supplemental Table S2. Meta-analysis Of Observational Studies in Epidemiology (MOOSE) Checklist

Supplemental Table S3. Modified Jadad Analysis for Methodological Quality

Supplemental Table S4. Funding Bias Scores for Studies

S3. Supplemental Figures

Supplemental Figure S1. Forest Plot showing Estimated Pooled Prevalence of sICH in AIS Patients with CMBs who Underwent Reperfusion Therapy

Supplemental Figure S2: Forest Plot showing Estimated Pooled Prevalence of sICH in AIS Patients with CMBs who Underwent IVT

Supplemental Figure S3. Forest Plot Showing Estimated Pooled Prevalence of HT In AIS Patients with CMBs who Underwent Reperfusion Therapy

Supplemental Figure S4. Forest Plot Showing Estimated Pooled Prevalence of Poor Functional Outcomes At 90 Days in AIS Patients with CMBs who Underwent Reperfusion Therapy

Supplemental Figure S5. Forest Plot Showing Estimated Pooled Prevalence of Mortality at 90 Days in AIS Patients with CMBs who Underwent Reperfusion Therapy

Supplemental Figure S6. Forest Plot of OR of sICH In AIS Patients with CMBs who Underwent Reperfusion Therapy

Supplemental Figure S7. Forest Plot of OR of sICH In AIS Patients with CMBs who Underwent IVT

Supplemental Figure S8. Forest Plot of OR of HT In AIS Patients with CMBs who Underwent Reperfusion Therapy

Supplemental Figure S9. Forest Plot of OR Of Poor Functional Outcome At 90 Days in AIS Patients with CMBs who Underwent Reperfusion Therapy.

Supplemental Figure S10. Forest Plot of OR Of 90 Day Mortality in AIS Patients with CMBs who Underwent Reperfusion Therapy

Supplemental Figure S11. Forest Plot of Risk Ratios (RR) of sICH in AIS Patients with CMBs who Underwent Reperfusion Therapy

Supplemental Figure S12. Forest Plot of Risk Ratios (RR) of sICH in AIS Patients with CMBs who Underwent IVT

Supplemental Figure S13. Forest Plot of RR of HT In AIS Patients with CMBs who Underwent Reperfusion Therapy
Supplemental Figure S14. Forest Plot of RR of Poor Functional Outcome at 90 Days in AIS Patients with CMBs who Underwent Reperfusion Therapy
Supplemental Figure S15. Forest Plot of RR of 90 Day Mortality in AIS Patients with CMBs who Underwent Reperfusion Therapy
Supplemental Figure S16. Egger's Test for The Meta-Analyses on the Association of CMBs in AIS Patients who Underwent Reperfusion Therapy with Various Clinical Outcomes
Supplemental Figure S17. Funnel Plots of Meta-Analyses on The Association Between Cmbs In AIS Patients Who Underwent Reperfusion Therapy With Various Clinical Outcomes
Supplemental Figure S18. Sensitivity Analyses for Meta-Analyses on the Association between CMBs in AIS patients and Various Clinical Outcomes following Reperfusion Therapy

S1. Search Strategy

PubMed

("cerebral microbleeds"[MeSH Terms] OR "microhemorrhages"[MeSH Terms] OR "microbleeds"[Title/Abstract] OR "microbleed"[Title/Abstract] OR "microbleedings"[Title/Abstract] OR "microhemorrhage"[Title/Abstract]) AND ("ischemic stroke"[MeSH Terms] OR "cerebrovascular ischemia"[MeSH Terms] OR "brain ischemia"[MeSH Terms] OR "stroke, acute"[MeSH Terms])

Filters applied: Clinical Study, Clinical Trial, Clinical Trial, Phase I, Clinical Trial, Phase II, Clinical Trial, Phase III, Clinical Trial, Phase IV, Comparative Study, Dataset, Evaluation Study, Multicenter Study, Observational Study, Randomised Controlled Trial, Validation Study, Humans, English, Adult: 19+ years.

Results: 53

Embase:

('cerebral microbleed'/exp OR 'microhemorrhage'/exp OR 'microbleed'/exp OR 'microbleeding'/exp OR 'microhemorrhagic stroke'/exp) AND ('ischemic stroke'/exp OR 'brain ischemia'/exp OR 'stroke'/exp)

('cerebral microbleed' OR 'microhemorrhage' OR 'microbleed' OR 'microbleeding' OR 'microhemorrhagic stroke') AND ('ischemic stroke' OR 'brain ischemia' OR 'stroke')

Results: 57

Cochrane

Title/Abstract: ("cerebral microbleeds" OR "microhemorrhages" OR "microbleeds" OR "microbleeding" OR "microhemorrhage") AND ("ischemic stroke" OR "cerebrovascular ischemia" OR "brain ischemia" OR "stroke, acute").

Results: 98

S2. List of Supplemental Tables

Supplemental Table S1. PRISMA 2020 Checklist

Section and Topic	Item #	Checklist item	Location where item is reported (Page)
**TITLE**			
Title	1	Identify the report as a systematic review.	1
**ABSTRACT**			
Abstract	2	See the PRISMA 2020 for Abstracts checklist.	2
**INTRODUCTION**			
Rationale	3	Describe the rationale for the review in the context of existing knowledge.	3
Objectives	4	Provide an explicit statement of the objective(s) or question(s) the review addresses.	3
**METHODS**			
Eligibility criteria	5	Specify the inclusion and exclusion criteria for the review and how studies were grouped for the syntheses.	3,4
Information sources	6	Specify all databases, registers, websites, organisations, reference lists and other sources searched or consulted to identify studies. Specify the date when each source was last searched or consulted.	3
Search strategy	7	Present the full search strategies for all databases, registers and websites, including any filters and limits used.	3-Supplementary Information
Selection process	8	Specify the methods used to decide whether a study met the inclusion criteria of the review, including how many reviewers screened each record and each report retrieved, whether they worked independently, and if applicable, details of automation tools used in the process.	3,4
Data collection process	9	Specify the methods used to collect data from reports, including how many reviewers collected data from each report, whether they worked independently, any processes for obtaining or confirming data from study investigators, and if applicable, details of automation tools used in the process.	4
Data items	10a	List and define all outcomes for which data were sought. Specify whether all results that were compatible with each outcome domain in each study were sought (e.g. for all measures, time points, analyses), and if not, the methods used to decide which results to collect.	4,5
	10b	List and define all other variables for which data were sought (e.g. participant and intervention characteristics, funding sources). Describe any assumptions made about any missing or unclear information.	4
Study risk of bias assessment	11	Specify the methods used to assess risk of bias in the included studies, including details of the tool(s) used, how many reviewers assessed each study and	4

Section and Topic	Item #	Checklist item	Location where item is reported (Page)
		whether they worked independently, and if applicable, details of automation tools used in the process.	
Effect measures	12	Specify for each outcome the effect measure(s) (e.g. risk ratio, mean difference) used in the synthesis or presentation of results.	4
Synthesis methods	13a	Describe the processes used to decide which studies were eligible for each synthesis (e.g. tabulating the study intervention characteristics and comparing against the planned groups for each synthesis (item #5)).	4
	13b	Describe any methods required to prepare the data for presentation or synthesis, such as handling of missing summary statistics, or data conversions.	N/A
	13c	Describe any methods used to tabulate or visually display results of individual studies and syntheses.	4
	13d	Describe any methods used to synthesize results and provide a rationale for the choice(s). If meta-analysis was performed, describe the model(s), method(s) to identify the presence and extent of statistical heterogeneity, and software package(s) used.	4
	13e	Describe any methods used to explore possible causes of heterogeneity among study results (e.g. subgroup analysis, meta-regression).	4
	13f	Describe any sensitivity analyses conducted to assess robustness of the synthesized results.	N/A
Reporting bias assessment	14	Describe any methods used to assess risk of bias due to missing results in a synthesis (arising from reporting biases).	4
Certainty assessment	15	Describe any methods used to assess certainty (or confidence) in the body of evidence for an outcome.	N/A
**RESULTS**			
Study selection	16a	Describe the results of the search and selection process, from the number of records identified in the search to the number of studies included in the review, ideally using a flow diagram.	5
	16b	Cite studies that might appear to meet the inclusion criteria, but which were excluded, and explain why they were excluded.	N/A
Study characteristics	17	Cite each included study and present its characteristics.	24
Risk of bias in studies	18	Present assessments of risk of bias for each included study.	Supplemental Table 3, 4

Section and Topic	Item #	Checklist item	Location where item is reported (Page)
Results of individual studies	19	For all outcomes, present, for each study: (a) summary statistics for each group (where appropriate) and (b) an effect estimate and its precision (e.g. confidence/credible interval), ideally using structured tables or plots.	31, 32
Results of syntheses	20a	For each synthesis, briefly summarise the characteristics and risk of bias among contributing studies.	31, 32
	20b	Present results of all statistical syntheses conducted. If meta-analysis was done, present for each the summary estimate and its precision (e.g. confidence/credible interval) and measures of statistical heterogeneity. If comparing groups, describe the direction of the effect.	31, 32
	20c	Present results of all investigations of possible causes of heterogeneity among study results.	N/A
	20d	Present results of all sensitivity analyses conducted to assess the robustness of the synthesized results.	N/A
Reporting biases	21	Present assessments of risk of bias due to missing results (arising from reporting biases) for each synthesis assessed.	N/A
Certainty of evidence	22	Present assessments of certainty (or confidence) in the body of evidence for each outcome assessed.	31,32
**DISCUSSION**			
Discussion	23a	Provide a general interpretation of the results in the context of other evidence.	7, 8, 9
	23b	Discuss any limitations of the evidence included in the review.	9
	23c	Discuss any limitations of the review processes used.	9
	23d	Discuss implications of the results for practice, policy, and future research.	9
**OTHER INFORMATION**			
Registration and protocol	24a	Provide registration information for the review, including register name and registration number, or state that the review was not registered.	N/A
	24b	Indicate where the review protocol can be accessed, or state that a protocol was not prepared.	N/A
	24c	Describe and explain any amendments to information provided at registration or in the protocol.	N/A
Support	25	Describe sources of financial or non-financial support for the review, and the role of the funders or sponsors in the review.	N/A
Competing interests	26	Declare any competing interests of review authors.	N/A

Section and Topic	Item #	Checklist item	Location where item is reported (Page)
Availability of data, code and other materials	27	Report which of the following are publicly available and where they can be found: template data collection forms; data extracted from included studies; data used for all analyses; analytic code; any other materials used in the review.	N/A

Sourced From: Page, M. J., McKenzie, J. E., Bossuyt, P. M., Boutron, I., Hoffmann, T. C., Mulrow, C. D., *et al*. The PRISMA 2020 statement: an updated guideline for reporting systematic reviews. *BMJ (Clinical research ed.)*, 2021; *372*, n71. https://doi.org/10.1136/bmj.n71.

Supplemental Table S2. MOOSE Checklist for Meta-analyses of Observational Studies

Item No	Recommendation	Reported on Page No
Reporting of background should include		
1	Problem definition	3
2	Hypothesis statement	3
3	Description of study outcome(s)	3
4	Type of exposure or intervention used	N/A
5	Type of study designs used	4
6	Study population	5
Reporting of search strategy should include		
7	Qualifications of searchers (eg, librarians and investigators)	N/A
8	Search strategy, including time period included in the synthesis and key words	Supplementary Information
9	Effort to include all available studies, including contact with authors	-
10	Databases and registries searched	3
11	Search software used, name and version, including special features used (eg, explosion)	3
12	Use of hand searching (eg, reference lists of obtained articles)	12-16
13	List of citations located and those excluded, including justification	5
14	Method of addressing articles published in languages other than English	-
15	Method of handling abstracts and unpublished studies	4, 5
16	Description of any contact with authors	N/A
Reporting of methods should include		
17	Description of relevance or appropriateness of studies assembled for assessing the hypothesis to be tested	4
18	Rationale for the selection and coding of data (eg, sound clinical principles or convenience)	4
19	Documentation of how data were classified and coded (eg, multiple raters, blinding and interrater reliability)	-

20	Assessment of confounding (eg, comparability of cases and controls in studies where appropriate)	-
21	Assessment of study quality, including blinding of quality assessors, stratification or regression on possible predictors of study results	-
22	Assessment of heterogeneity	31
23	Description of statistical methods (eg, complete description of fixed or random effects models, justification of whether the chosen models account for predictors of study results, dose-response models, or cumulative meta-analysis) in sufficient detail to be replicated	3
24	Provision of appropriate tables and graphics	18-32
Reporting of results should include		
25	Graphic summarising individual study estimates and overall estimate	31, 32
26	Table giving descriptive information for each study included	24, 25
27	Results of sensitivity testing (eg, subgroup analysis)	N/A
28	Indication of statistical uncertainty of findings	-
**Item No**	**Recommendation**	**Reported on Page No**
Reporting of discussion should include		
29	Quantitative assessment of bias (eg, publication bias)	31, 32
30	Justification for exclusion (eg, exclusion of non-English language citations)	4
31	Assessment of quality of included studies	7-Supplemental Information Table 1
Reporting of conclusions should include		
32	Consideration of alternative explanations for observed results	8, 9, 10
33	Generalisation of the conclusions (ie, appropriate for the data presented and within the domain of the literature review)	10
34	Guidelines for future research	9
35	Disclosure of funding source	N/A

Sourced From: Stroup DF, Berlin JA, Morton SC, et al, for the Meta-analysis Of Observational Studies in Epidemiology (MOOSE) Group. Meta-analysis of Observational Studies in Epidemiology. A Proposal for Reporting. *JAMA*. 2000;283(15):2008-2012. doi: 10.1001/jama.283.15.2008.

Supplemental Table S3. Modified Jadad Analysis

Author	Criteria 1[a]	Criteria 2[b]	Criteria 3[c]	Criteria 4[d]	Criteria 5[e]	Criteria 6[f]	Criteria 7[g]	Criteria 8[h]	Total
Bai et al.	0	0	0	0	1	1	1	1	4
Braemswig et al.	1	1	1	1	1	1	1	1	8
Brauner et al.	1	1	1	1	1	1	1	1	8
Chacon-Portillo et al	0	0	0	0	1	1	1	1	4
Chatzikonstaninou et al.	0	0	0	0	1	1	1	1	4

Choi et al (1)	0	0	0	0	1	1	1	1	4
Choi et al (2)	0	0	0	0	1	1	1	1	4
Dannenburg et al	0	0	0	0	1	1	1	1	4
Derex et al	0	0	0	0	1	1	1	1	4
Derraz et al.	0	0	0	0	1	1	1	1	4
Fiehler et al.	0	0	0	0	1	1	1	1	4
Gratz et al.	0	0	0	0	0	1	1	1	3
Kakuda et al.	0	0	0	0	1	1	1	1	4
Kidwell et al.	0	0	0	0	0	1	1	1	3
Kim et al.	0	0	0	0	1	1	1	1	4
Kimura et al.	0	0	0	0	1	1	1	1	4
Lee et al.	0	0	0	0	1	1	1	1	4
Moriya et al.	0	0	0	0	1	1	1	1	4
Nighoghossian et al.	1	0	0	0	1	1	1	1	4
Pratz-Sanchez et al.	0	0	0	0	1	1	1	1	4
Schlemm et al.	0	0	0	0	1	1	1	1	4
Shi et al.	0	0	0	0	1	1	1	1	4
Soo et al.	0	0	0	0	1	1	1	1	4
Turc et al.	0	0	0	0	1	1	1	1	4
Yan et al.	0	0	0	0	1	1	1	1	4
Zand et al.	0	0	0	0	1	1	1	1	4

N.B. no = 0, yes = 1, Total = sum of scores from criteria 1-8. [a]: Criteria 1: was the study randomised? [b]: Criteria 2: was the method of randomisation appropriate? [c]: Criteria 3: was the study described as being blinded? [d]: Criteria 4: was the method of blinding appropriate (single/partially blinded = 0.5) [e]: Criteria 5: was there a description of withdrawals and dropouts? [f]: Criteria 6: was there a clear description of the inclusion/exclusion criteria? [g]: Criteria 7: was the method used to assess adverse events described? [h]: Criteria 8: was the method of statistical analysis described?

Supplemental Table S4: Funding Bias Scores for Studies

**Author**	**Funding Bias**
Bai et al.	0
Brauner et al.	1
Chacon-Portillo et al	0
Chatzikonstaninou et al	0
Choi et al	0
Choi et al (2)	0
Dannenburg et al	1
Derex et al.	0
Derraz et al.	0
Fiehler et al.	0
Gratz et al.	0
Kakuda et al.	0

Kidwell et al.	2
Kim et al.	0
Kimura et al.	0
Lee et al.	0
Moriya et al.	0
Nighoghossian et al.	0
Pratz-Sanchez et al.	0
Schlemm et al.	0
Shi et al.	0
Soo et al.	0
Turc et al.	0
Yan et al.	0
Zand et al.	0

N.B. 0 = low potential for bias, 1 = any conflicts of interest declared relating to industry funding outside of the current research publication, 2 = study funded by industry, 3 = high potential for bias.

S3. List of Supplemental Figures

Supplemental Figure S1. Forest plot showing estimated pooled prevalence of sICH in AIS patients with CMBs undergoing Reperfusion Therapy

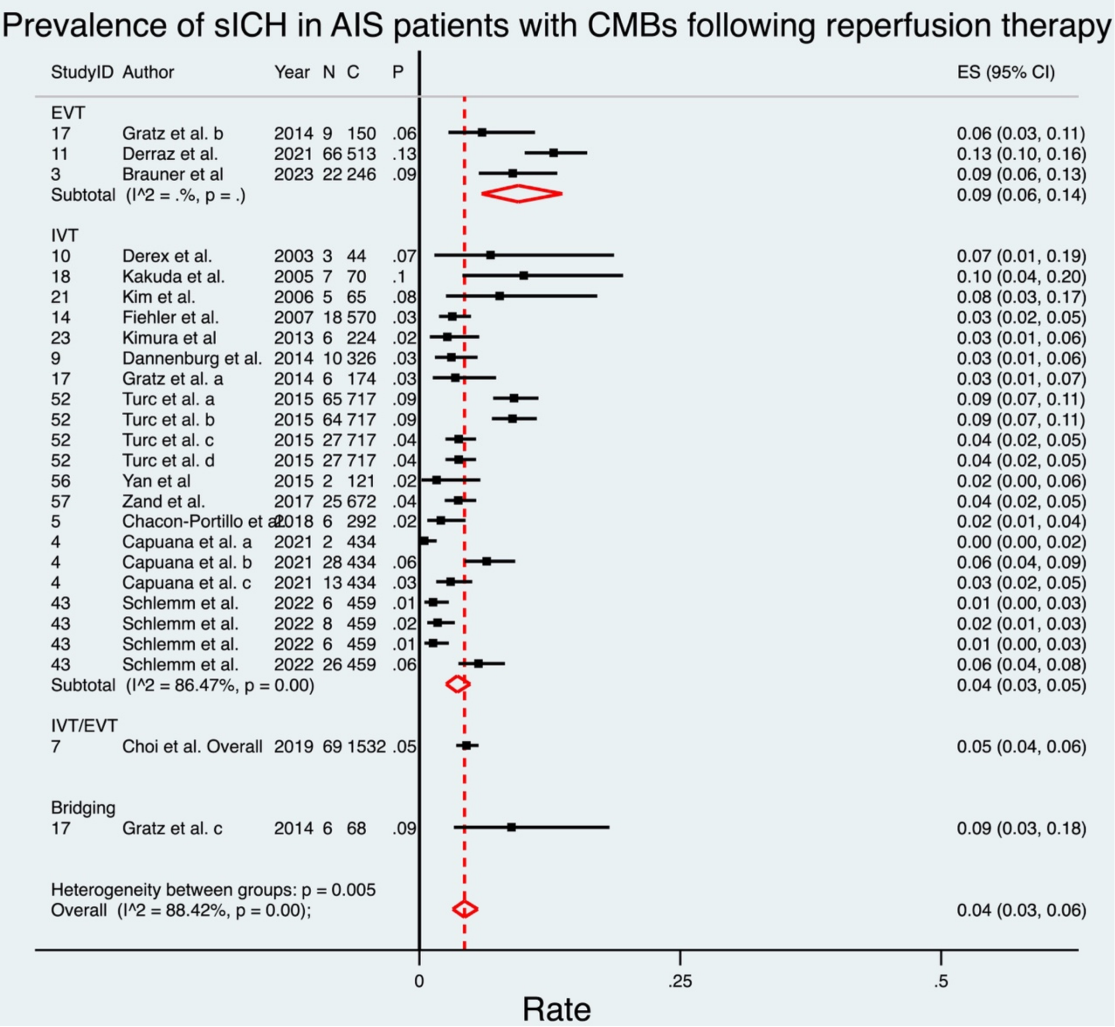

Abbreviations: CMB= cerebral microbleeds, AIS= acute ischaemic stroke, N= number of patients with CMBs, C= total cohort number, P= prevalence, sICH = symptomatic intracerebral haemorrhage, IVT = intravenous thrombolysis, EVT = endovascular thrombectomy, CI = confidence interval, ES= effect size, I^2= heterogeneity value, p= p-value.

Supplemental Figure S2. Forest plot showing estimated pooled prevalence of sICH in AIS patients with CMBs who underwent IVT

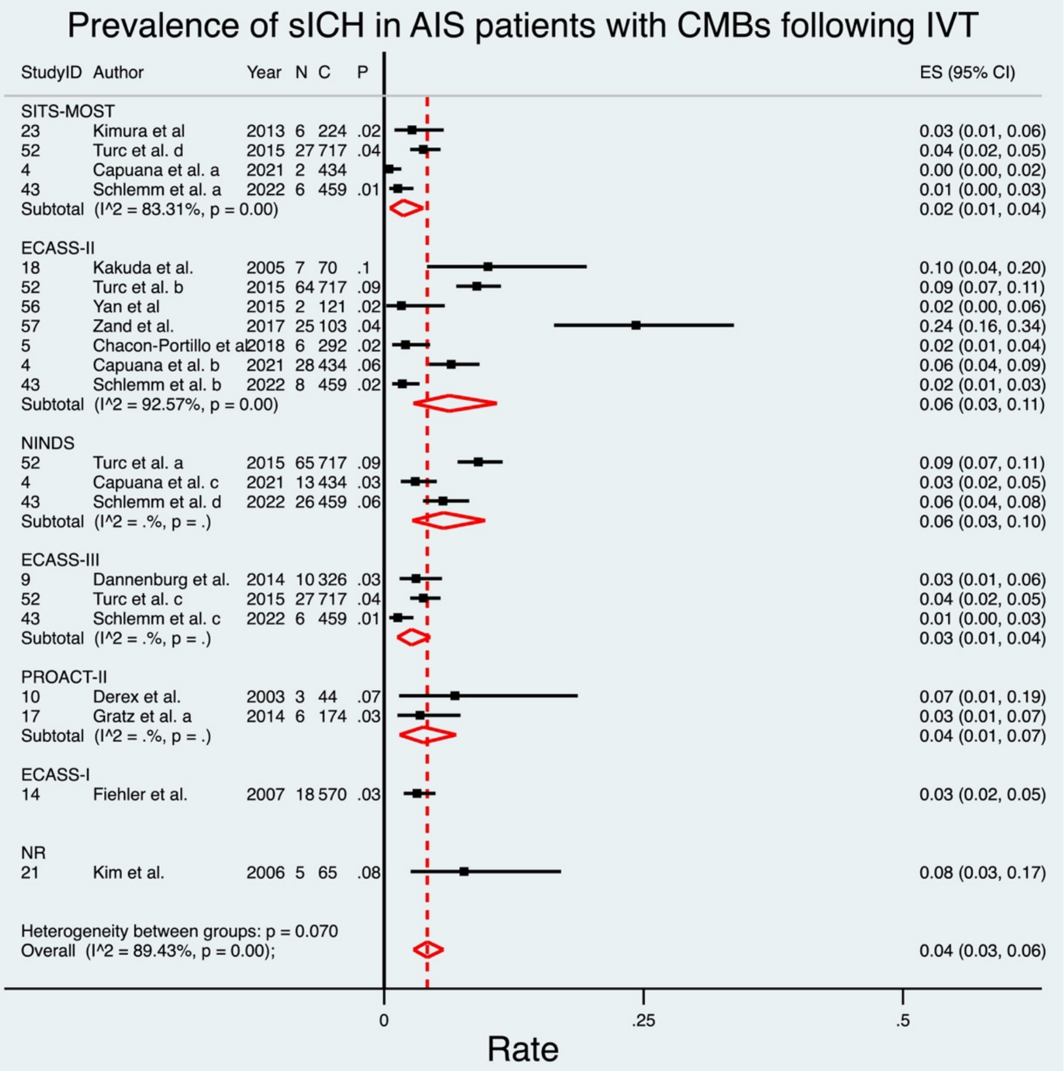

Abbreviations: CMB= cerebral microbleeds, AIS= acute ischaemic stroke, N= number of patients with CMBs, C= total cohort number, P= prevalence, ECASS-I= first European Cooperative Acute Stroke Study, ECASS-II= second European Cooperative Acute Stroke Study, ECASS-III= third European Cooperative Acute Stroke Study, NINDS= National Institute of Neurological Disorders and Stroke, SITS-MOST= Safe Implementation of Thrombolysis in Stroke-Monitoring Study, PROACT-II= Prolyse in Acute Cerebral Thromboembolism trial, NR= not reported, sICH= symptomatic intracerebral haemorrhage, IVT = intravenous thrombolysis, NR = not reported, ES= effect size, I^2= heterogeneity value, p= p-value.

Supplemental Figure S3. Forest plot showing estimated pooled prevalence of HT in AIS patients with CMBs who underwent reperfusion therapy

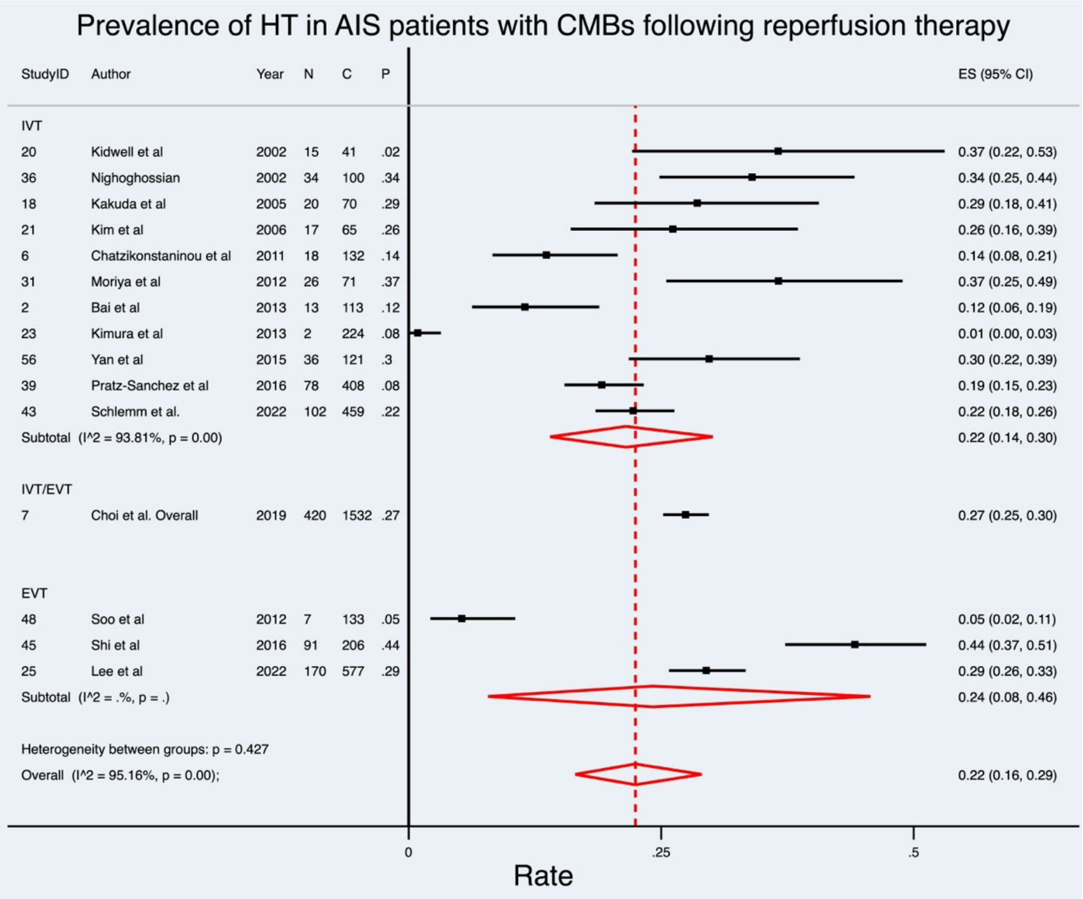

Abbreviations: CMB= cerebral microbleeds, AIS= acute ischaemic stroke, N= number of patients with CMBs, C= total cohort number, P= prevalence, HT = haemorrhagic transformation, IVT = intravenous thrombolysis, EVT = endovascular thrombectomy, CI = confidence interval, ES= effect size, I^2= heterogeneity value, p= p-value.

Supplemental Figure S4. Forest plot showing estimated pooled prevalence of poor functional outcomes at 90 days in AIS patients with CMBs who underwent reperfusion therapy

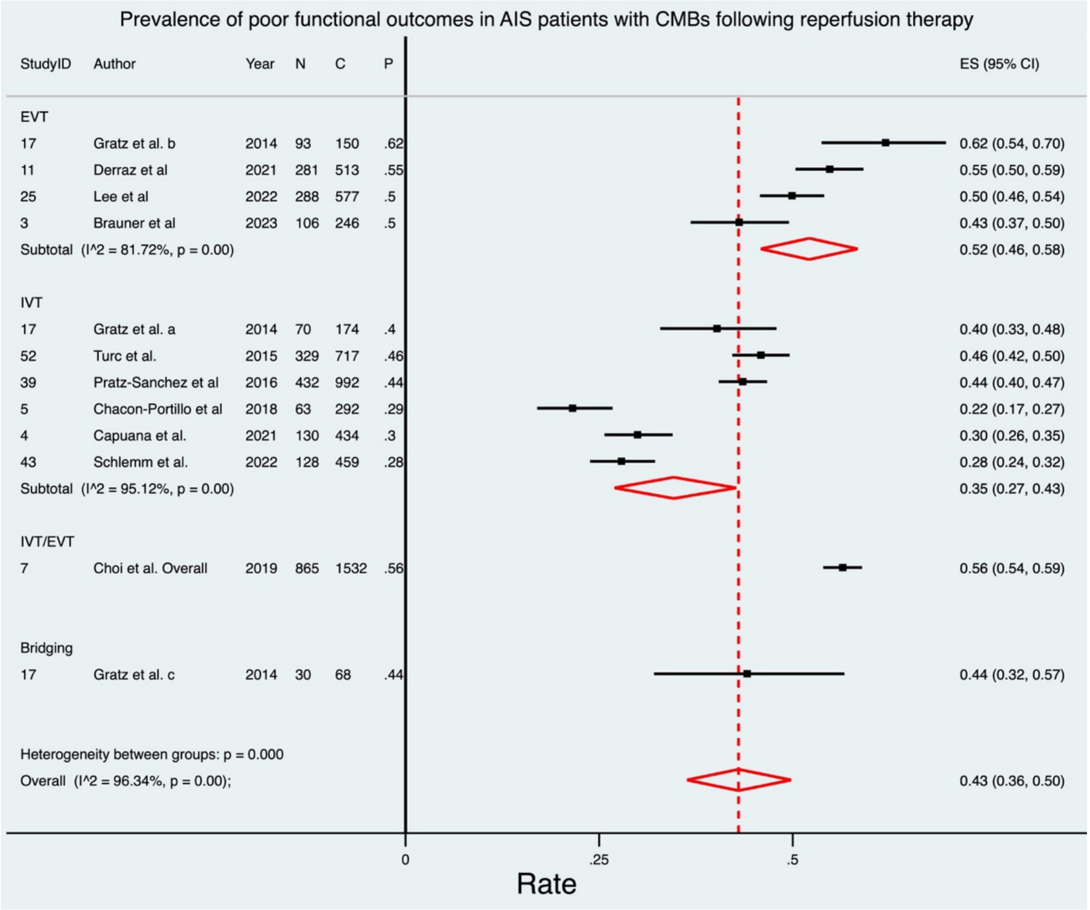

Abbreviations: CMB= cerebral microbleeds, AIS= acute ischaemic stroke, N= number of patients with CMBs, C= total cohort number, P= prevalence, IVT = intravenous thrombolysis, EVT = endovascular thrombectomy, CI = confidence interval, ES= effect size, I^2= heterogeneity value, p= p-value.

Supplemental Figure S5. Forest plot showing estimated pooled prevalence of mortality at 90 days in AIS patients with CMBs who underwent reperfusion therapy

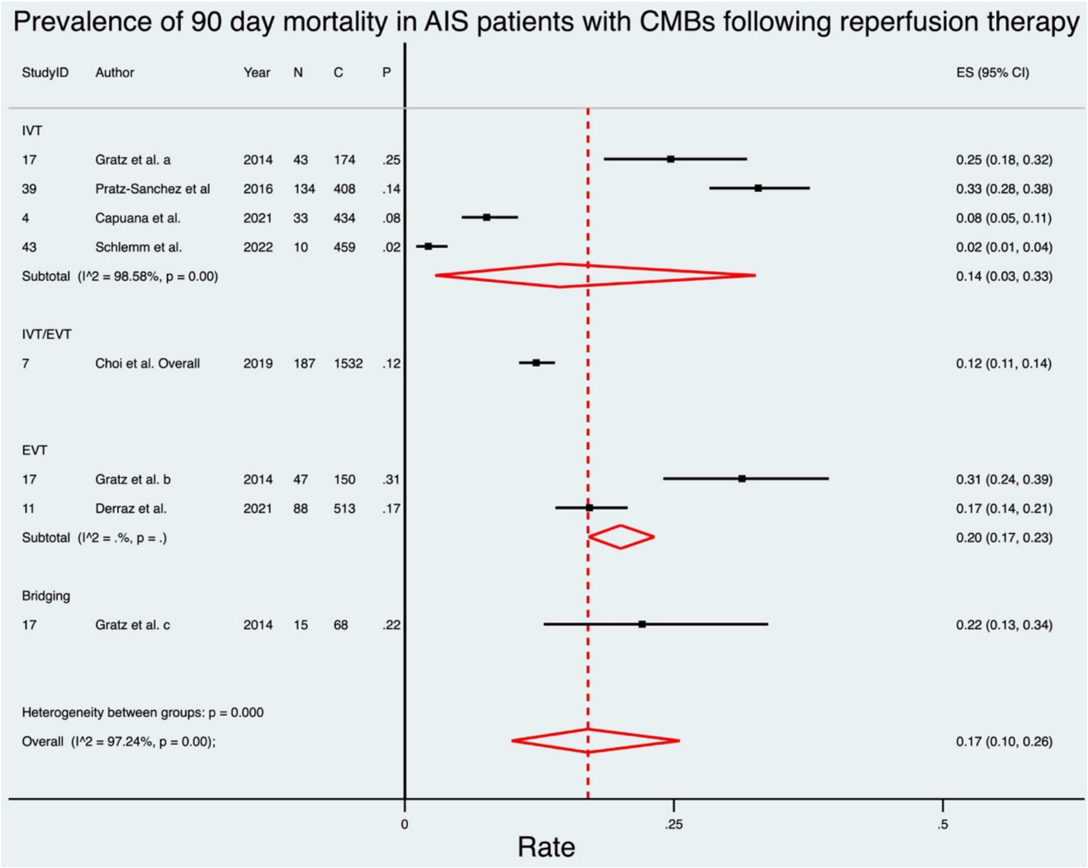

Abbreviations: CMB= cerebral microbleeds, AIS= acute ischaemic stroke, N= number of patients with CMBs, C= total cohort number, P= prevalence, IVT = intravenous thrombolysis, EVT = endovascular thrombectomy, CI = confidence interval, ES= effect size, I^2= heterogeneity value, p= p-value.

Supplemental Figure S6. Forest plot of Odds Ratios (OR) of sICH in AIS patients with CMBs who underwent Reperfusion Therapy

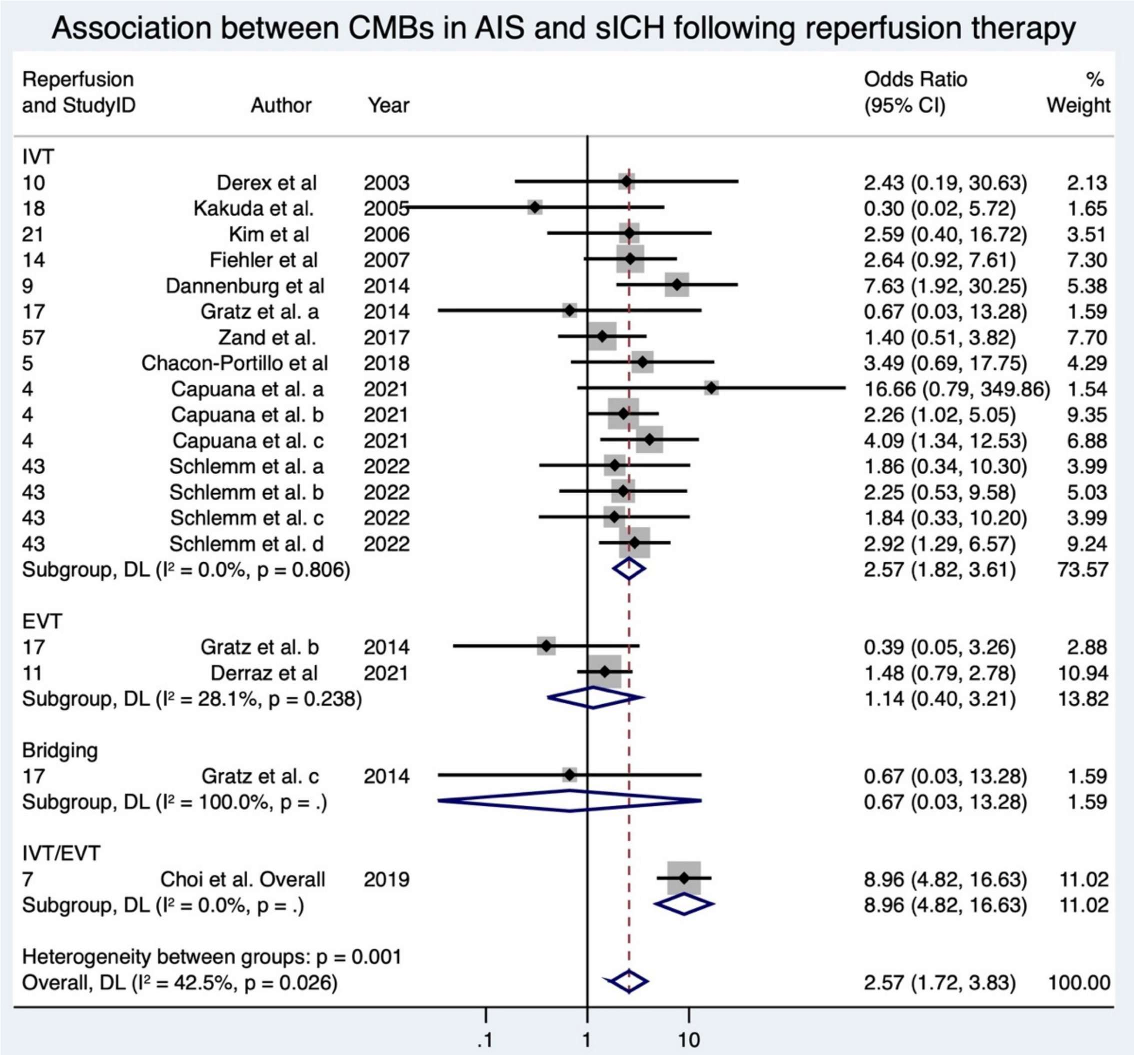

Abbreviations: CMBs= cerebral microbleeds, AIS = acute ischaemic stroke, sICH= symptomatic intracerebral haemorrhage, IVT= intravenous thrombolysis, EVT= endovascular thrombectomy, OR = odds ratio, CI= confidence interval, p= p-value, DL= DerSimmonian and Laird, I^2= heterogeneity.

Supplemental Figure S7. Forest plot of Odds Ratios (OR) of sICH in AIS patients with CMBs who underwent IVT

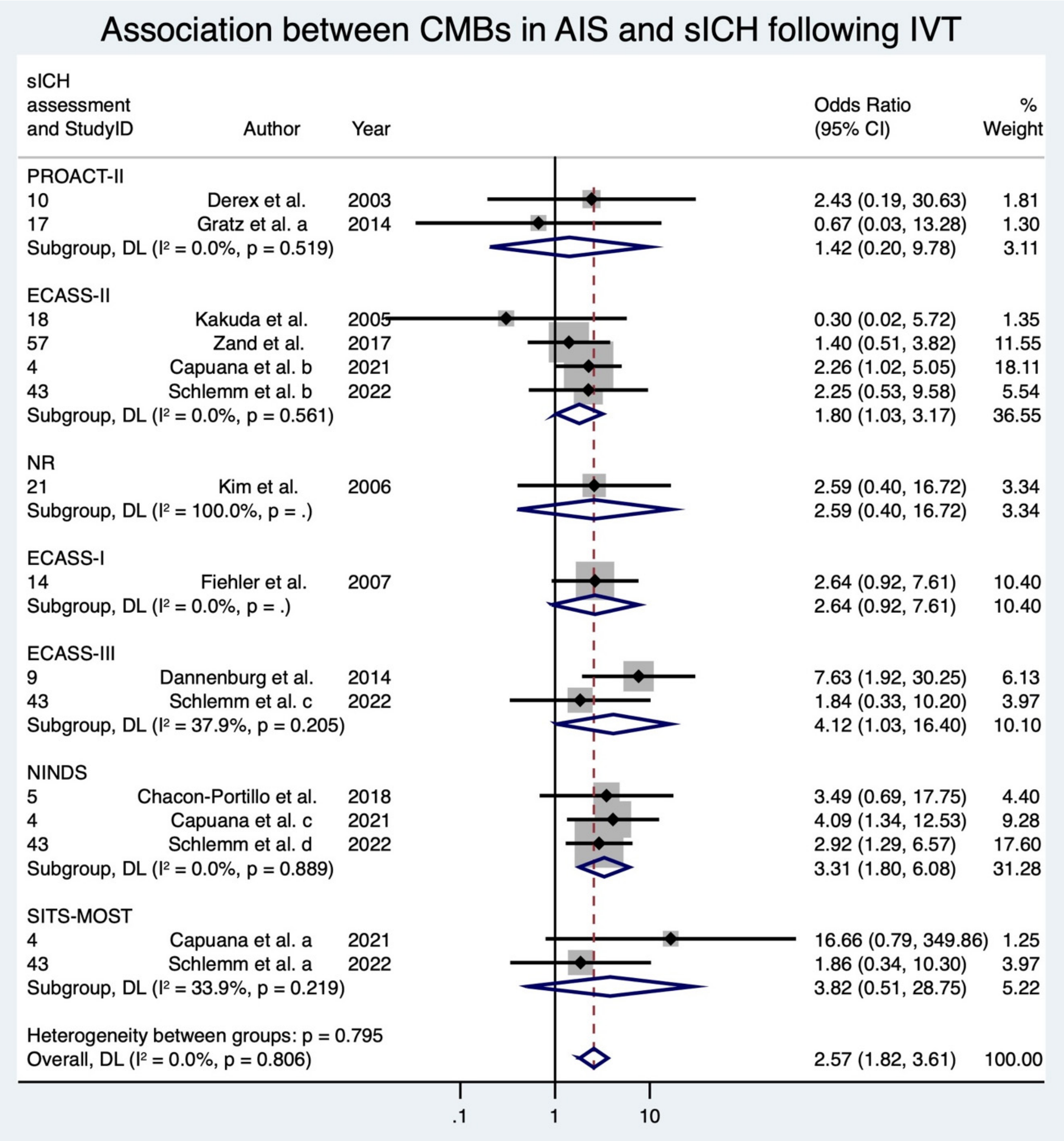

Abbreviations: CMBs= cerebral microbleeds, AIS = acute ischaemic stroke, sICH= symptomatic intracerebral haemorrhage, IVT= intravenous thrombolysis, PROACT-II= Prolyse in Acute Cerebral Thromboembolism trial 2, ECASS-I= first European Cooperative Acute Stroke Study, ECASS-II= second European Cooperative Acute Stroke Study, ECASS-III= third European Cooperative Acute Stroke Study, NINDS= National Institute of Neurological Disorders and Stroke, SITS-MOST= Safe Implementation of Thrombolysis in Stroke-Monitoring Study, NR= not reported, OR = odds ratio, CI= confidence interval, p= p-value, DL= DerSimmonian and Laird, I^2= heterogeneity.

Supplemental Figure S8. Forest plot of OR of HT in AIS patients with CMBs who underwent reperfusion therapy

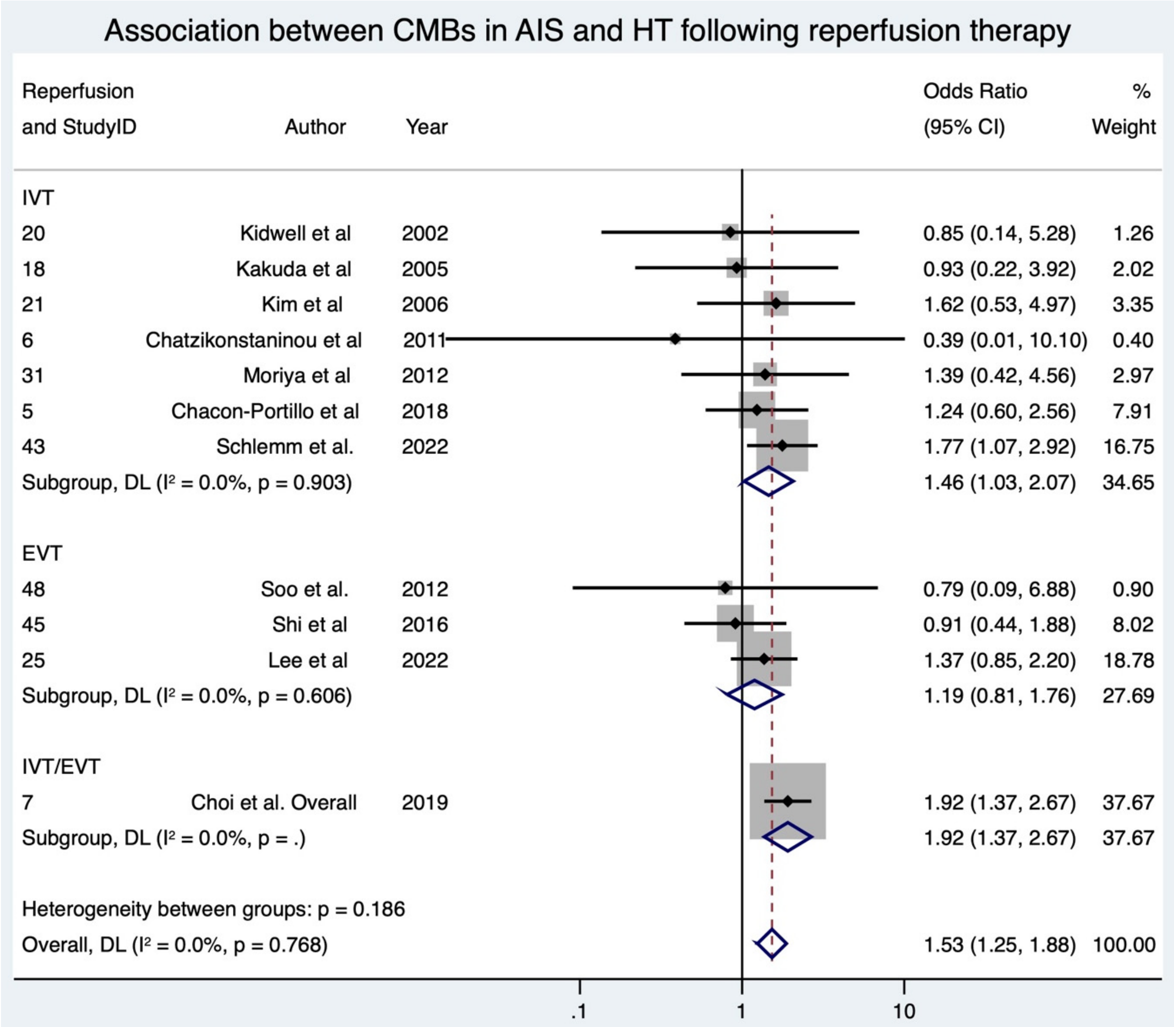

Abbreviations: CMBs= cerebral microbleeds, sICH= symptomatic intracerebral haemorrhage, AIS = acute ischaemic stroke, HT= haemorrhagic transformation, IVT= intravenous thrombolysis, EVT= endovascular thrombectomy, IVT/EVT= IVT or EVT, OR = odds ratio, CI= confidence interval, p= p-value, DL= DerSimmonian and Laird, I^2= heterogeneity.

Supplemental Figure S9. Forest plot of OR of poor functional outcome at 90 days in AIS patients with CMBs who underwent reperfusion therapy.

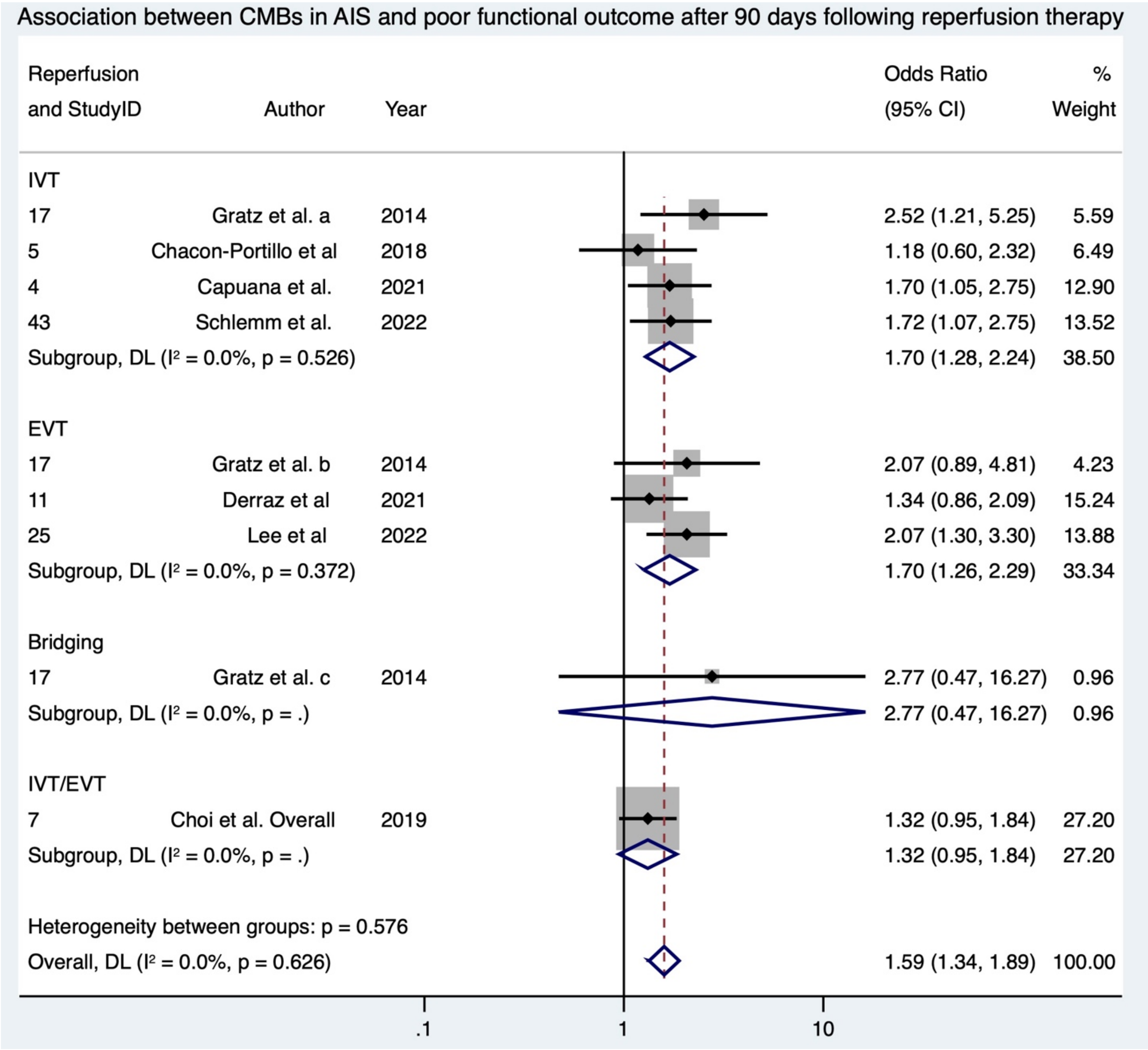

Abbreviations: CMBs= cerebral microbleeds, AIS = acute ischaemic stroke, IVT= intravenous thrombolysis, EVT= endovascular thrombectomy, IVT/EVT= IVT or EVT OR = odds ratio, CI= confidence interval, p= p-value, DL= DerSimmonian and Laird, I^2= heterogeneity.

Supplemental Figure S10. Forest plot of OR of 90 day mortality in AIS patients with CMBs who underwent reperfusion therapy

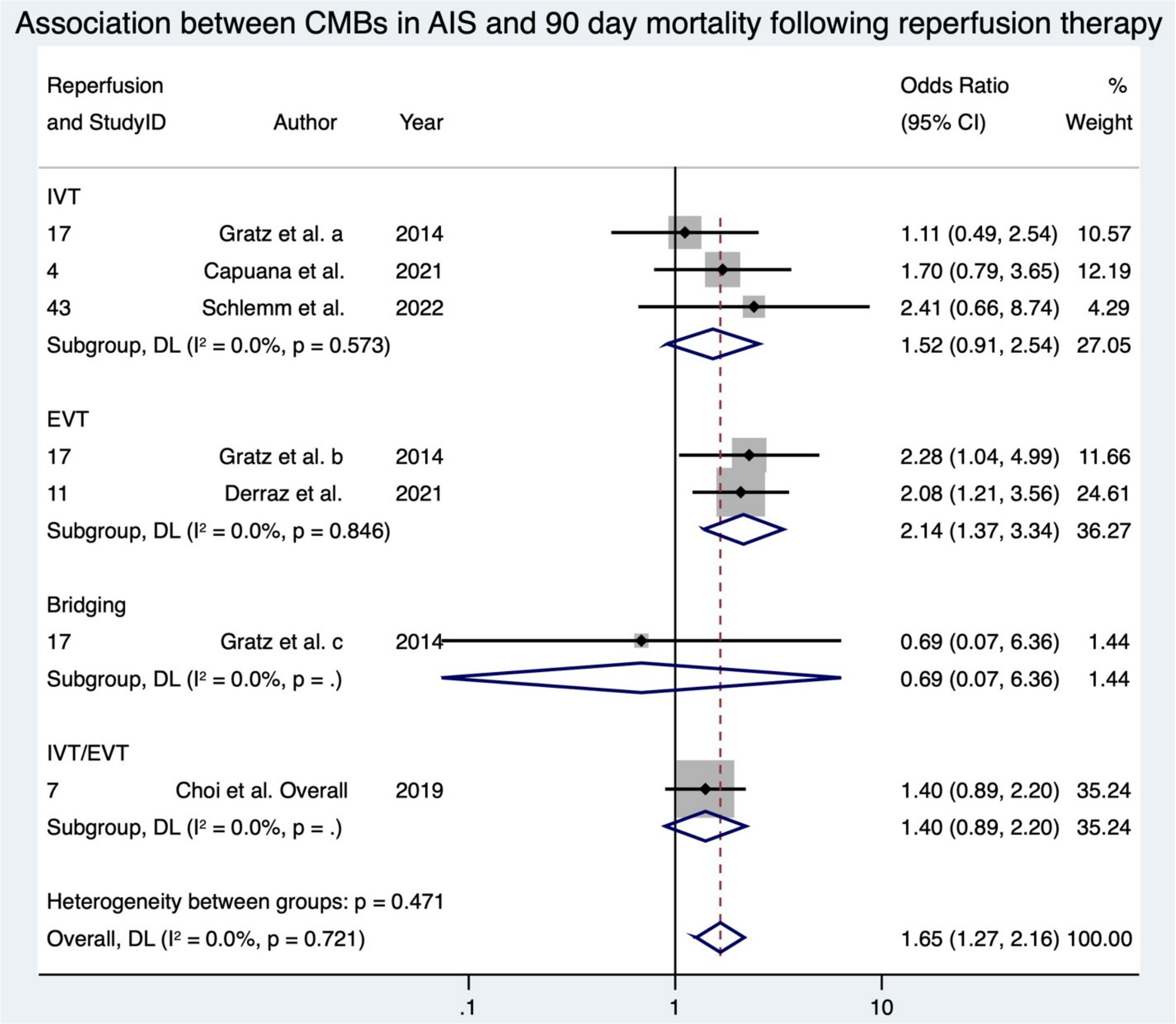

Abbreviations: CMBs= cerebral microbleeds, AIS = acute ischaemic stroke, IVT= intravenous thrombolysis, EVT= endovascular thrombectomy, IVT/EVT= IVT or EVT, OR = odds ratio, CI= confidence interval, p= p-value, DL= DerSimmonian and Laird, I^2= heterogeneity.

Supplemental Figure S11. Forest plot of Risk Ratios (RR) of sICH in AIS patients with CMBs who underwent Reperfusion Therapy

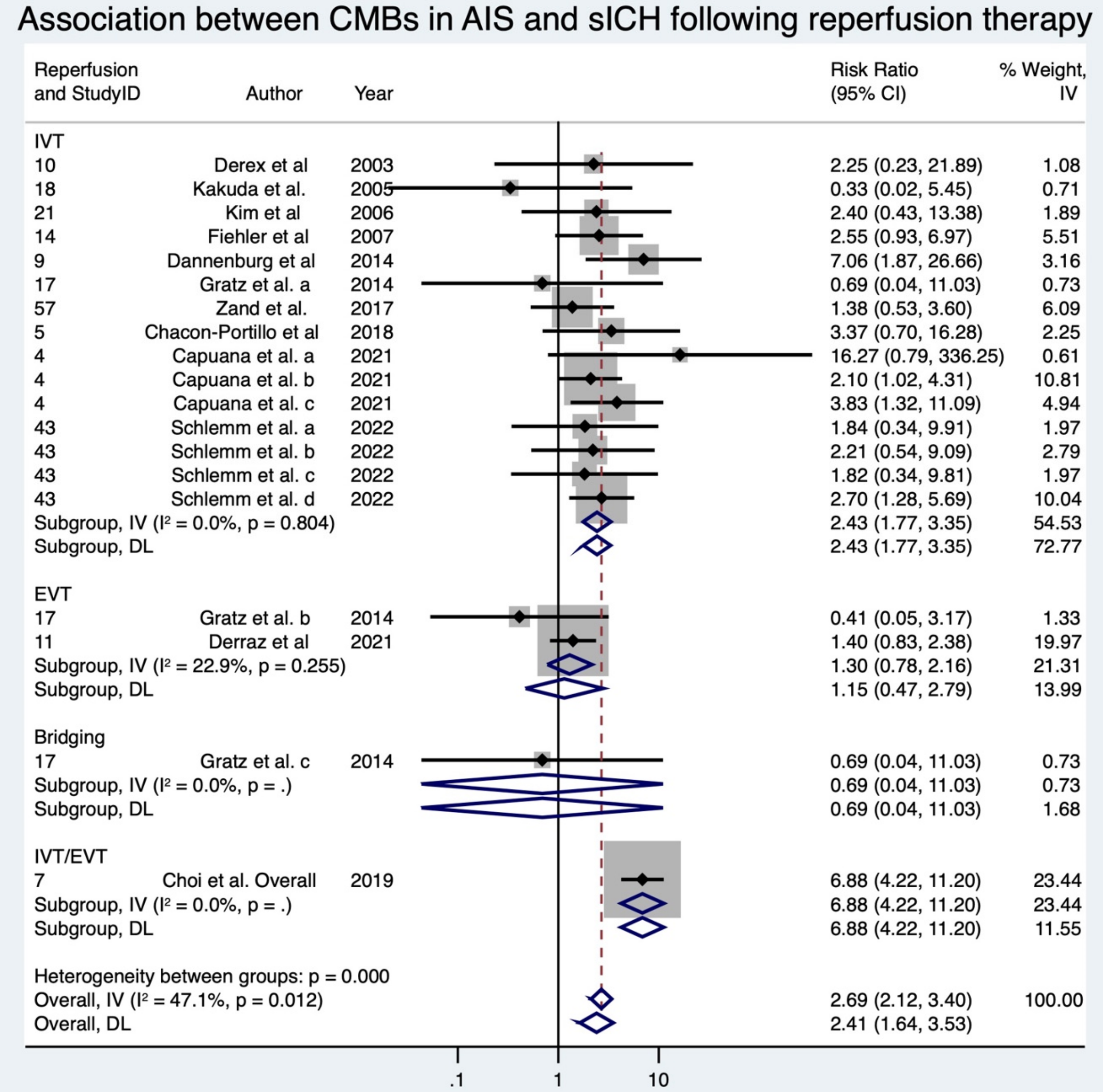

Abbreviations: CMBs= cerebral microbleeds, AIS = acute ischaemic stroke, IVT= intravenous thrombolysis, EVT= endovascular thrombectomy, IVT/EVT= IVT or EVT, RR = risk ratio, CI= confidence interval, p= p-value, DL= DerSimmonian and Laird, I^2= heterogeneity.

Supplemental Figure S12. Forest plot of Risk Ratios (RR) of sICH in AIS patients with CMBs who underwent IVT

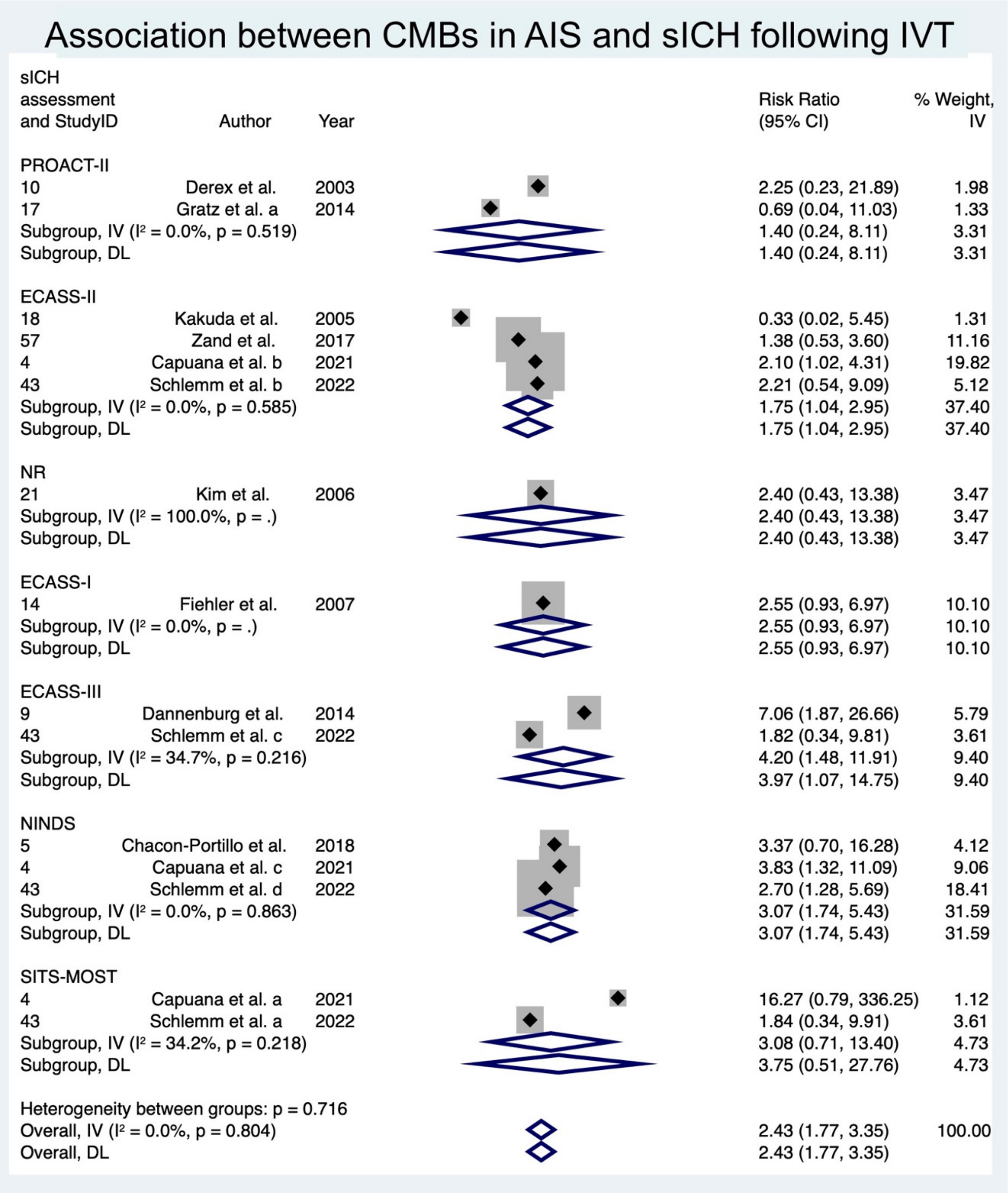

Abbreviations: CMBs= cerebral microbleeds, AIS = acute ischaemic stroke, sICH= symptomatic intracerebral haemorrhage, IVT= intravenous thrombolysis, PROACT-II= Prolyse in Acute Cerebral Thromboembolism trial 2, ECASS-I= first European Cooperative Acute Stroke Study, ECASS-II= second European Cooperative Acute Stroke Study, ECASS-III= third European Cooperative Acute Stroke Study, NINDS= National Institute of Neurological Disorders and Stroke, SITS-MOST= Safe Implementation of Thrombolysis in Stroke-Monitoring Study, NR= not reported, RR = risk ratio, CI= confidence interval, p= p-value, DL= DerSimmonian and Laird, I^2= heterogeneity.

Supplemental Figure S13. Forest plot of RR of HT in AIS patients with CMBs who underwent reperfusion therapy

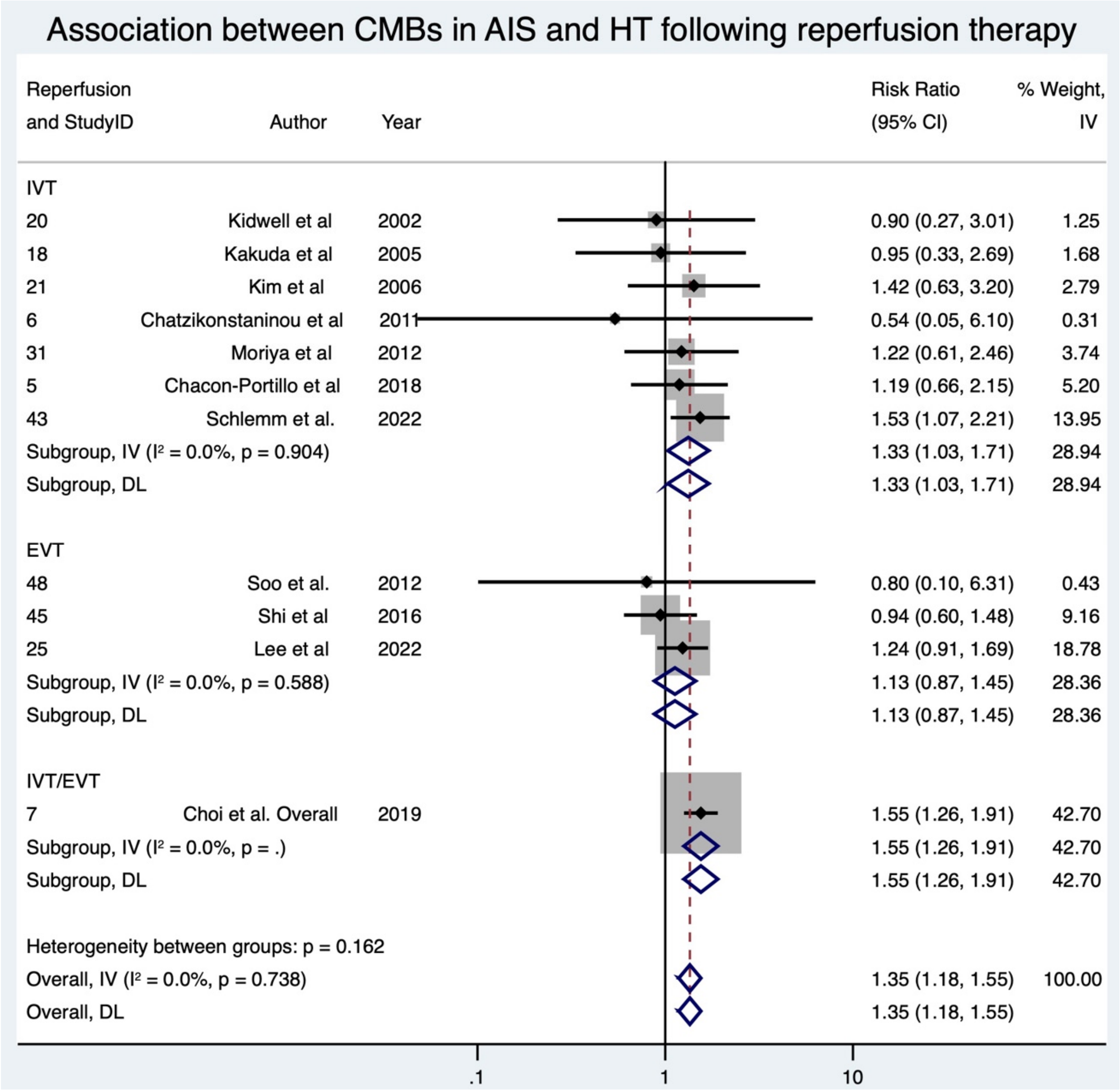

Abbreviations: CMBs= cerebral microbleeds, AIS = acute ischaemic stroke, HT= haemorrhagic transformation, IVT= intravenous thrombolysis, EVT= endovascular thrombectomy, IVT/EVT= IVT or EVT, RR = risk ratio, CI= confidence interval, p= p-value, DL= DerSimmonian and Laird, I^2= heterogeneity.

Supplemental Figure S14. Forest plot of RR of poor functional outcome at 90 days in AIS patients with CMBs who underwent reperfusion therapy

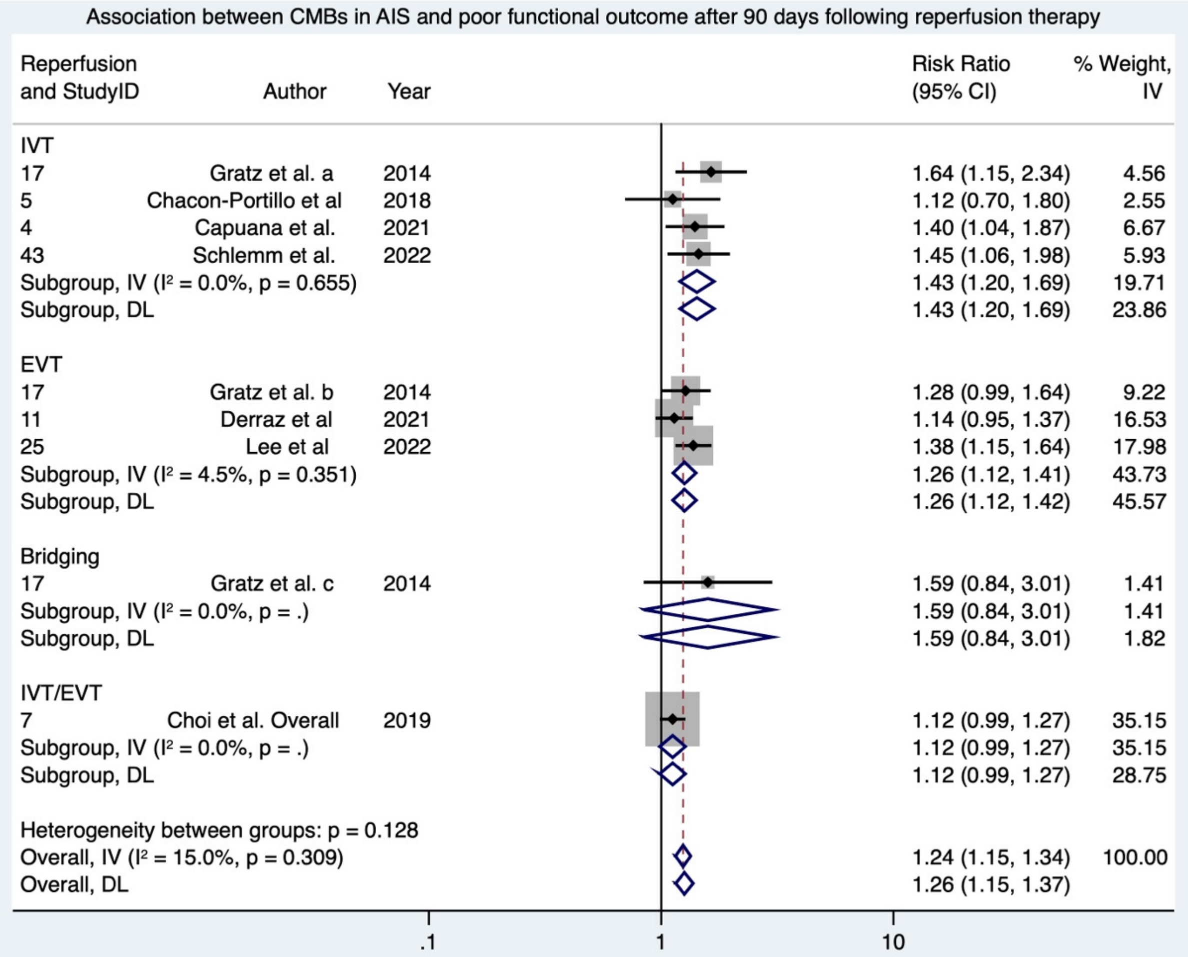

Abbreviations: CMBs= cerebral microbleeds, AIS= acute ischaemic stroke, IVT= intravenous thrombolysis, EVT= endovascular thrombectomy, IVT/EVT= IVT or EVT, RR = risk ratio, CI= confidence interval, p= p-value, DL= DerSimmonian and Laird, I^2= heterogeneity.

Supplemental Figure S15. Forest plot of RR of 90 day mortality in AIS patients with CMBs who underwent reperfusion therapy

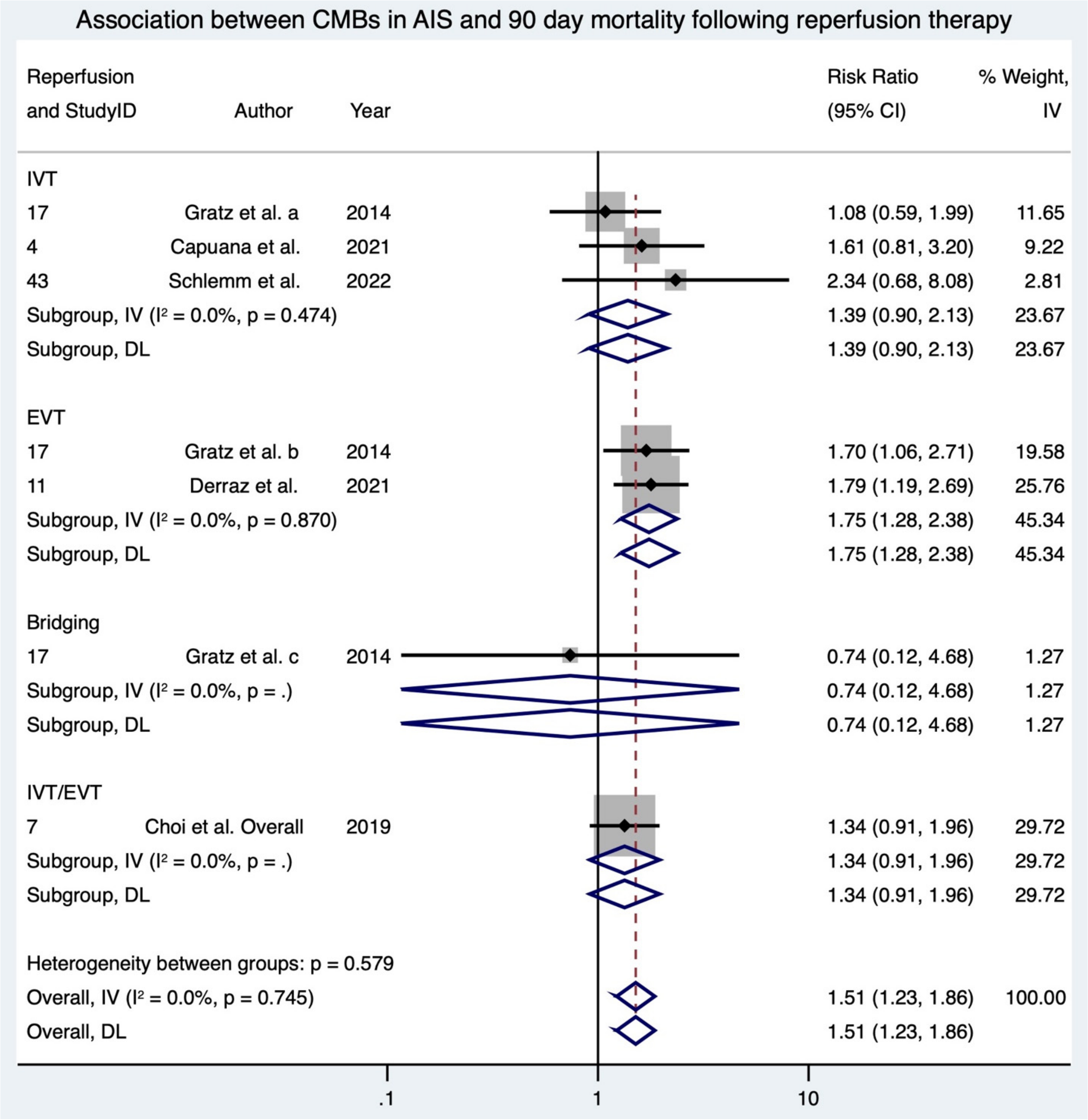

Abbreviations: CMBs= cerebral microbleeds, AIS = acute ischaemic stroke, IVT= intravenous thrombolysis, EVT= endovascular thrombectomy, IVT/EVT= IVT or EVT, RR = risk ratio, CI= confidence interval, p= p-value, DL= DerSimmonian and Laird, I^2= heterogeneity.

Supplemental Figure S16. Egger's test for the meta-analyses on the association of CMBs in AIS patients who underwent reperfusion therapy with various clinical outcomes

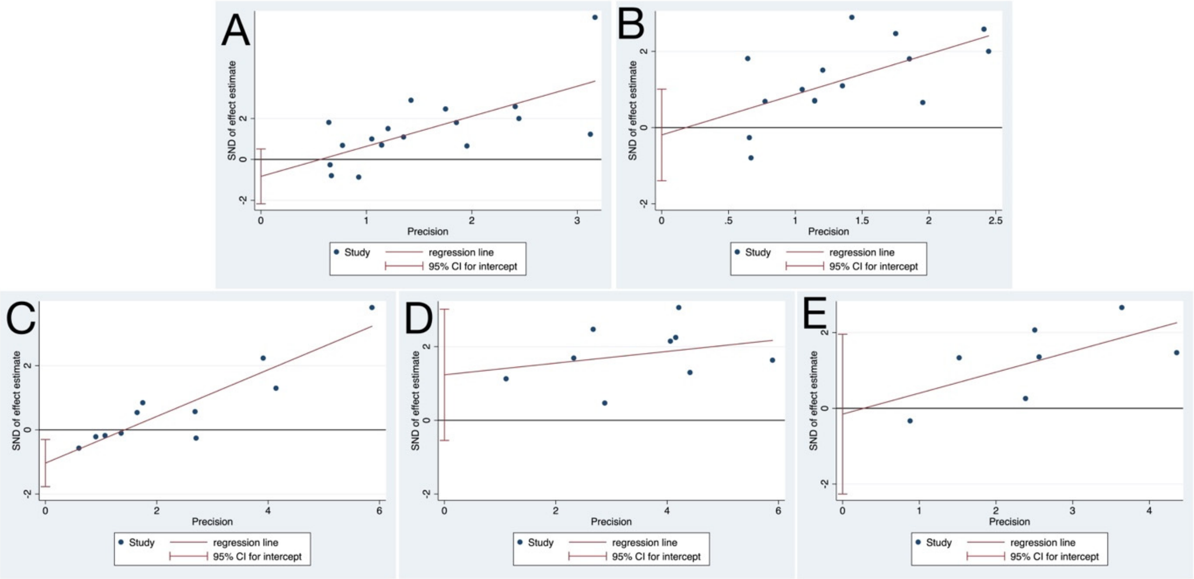

Scheme 16. A. Egger's test of sICH in AIS patients with CMBs who underwent reperfusion therapy. **B.** Egger's test of sICH in AIS patients with CMBs who underwent IVT only. **C.** Egger's test of HT in AIS patients with CMBs who underwent reperfusion therapy. **D.** Egger's test of poor functional outcome at 90 days in AIS patients with CMBs who underwent reperfusion therapy. **E.** Egger's test of 90-day mortality in AIS patients with CMBs who underwent reperfusion therapy. Abbreviations: CI = confidence interval, SND = standard normal deviate, CMB = cerebral microbleed, AIS = acute ischaemic stroke, IVT = intravenous thrombolysis, sICH = symptomatic intracerebral haemorrhage, HT = haemorrhagic transformation.

Supplemental Figure S17. Funnel plots of meta-analyses on the association between CMBs in AIS who underwent reperfusion therapy with various clinical outcomes

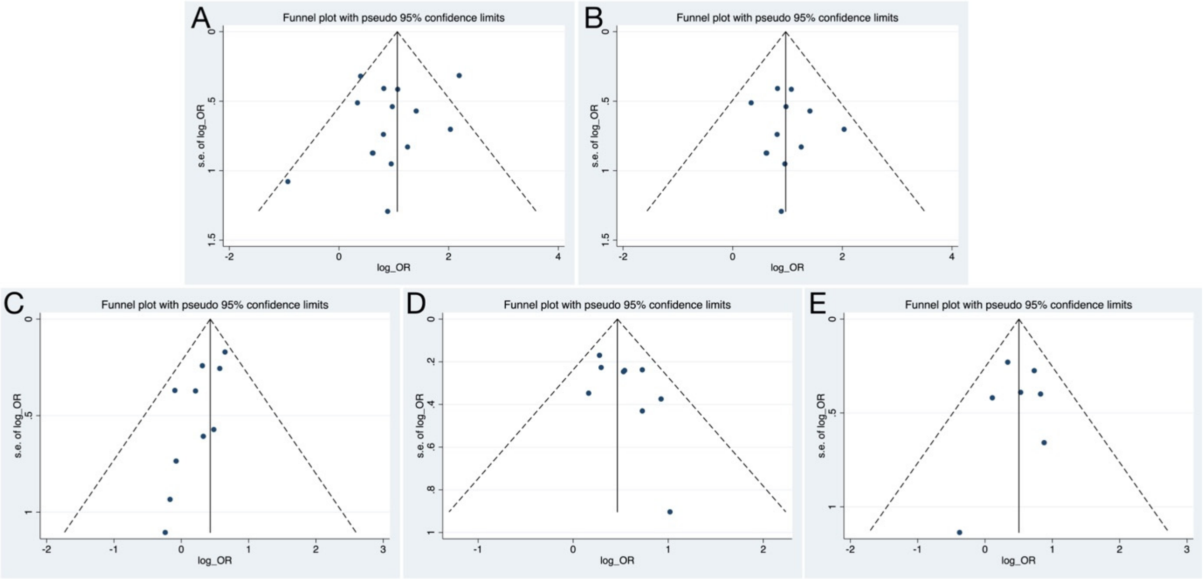

A. Funnel plot for publication bias of sICH in AIS patients with CMBs who underwent reperfusion therapy. **B.** Funnel plot for publication bias of sICH in AIS patients with CMBs who underwent IVT only. **C.** Funnel plot for publication bias of HT in AIS patients with CMBs who underwent reperfusion therapy. **D.** Funnel plot for publication bias of poor functional outcome at 90 days in AIS patients with CMBs who underwent reperfusion therapy. **E.** Funnel plot for publication bias of 90 day

mortality in AIS patients with CMBs who underwent reperfusion therapy. Abbreviations: OR= odds ratio, s.e.= standard error, CMB = cerebral microbleed, AIS = acute ischaemic stroke, IVT = intravenous thrombolysis, sICH = symptomatic intracerebral haemorrhage, HT = haemorrhagic transformation.

Supplemental Figure S18. Sensitivity Analyses for Meta-Analyses on the Association between CMBs in AIS patients and Various Clinical Outcomes following Reperfusion Therapy

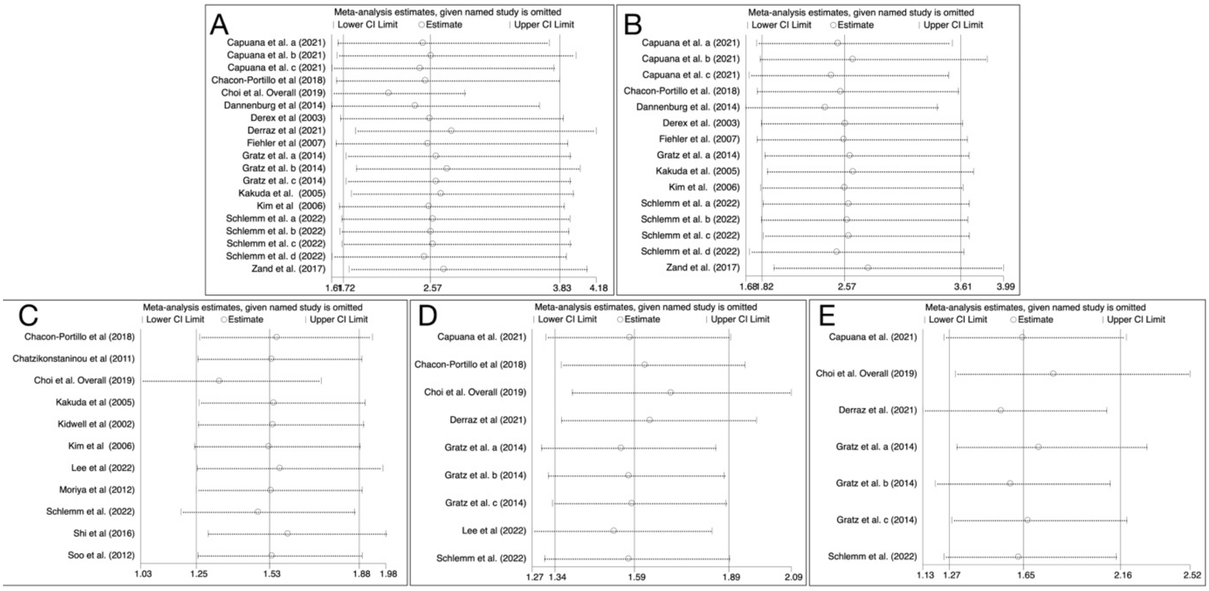

A. Sensitivity Analyses for Meta-Analyses on the Association between sICH and CMBs in AIS patients who underwent reperfusion therapy. **B.** Sensitivity Analyses for Meta-Analyses on the Association between sICH and CMBs in AIS patients who underwent IVT only. **C.** Sensitivity Analyses for Meta-Analyses on the Association between HT and CMBs in AIS patients who underwent reperfusion therapy. **D.** Sensitivity Analyses for Meta-Analyses on the Association between Poor Functional Outcome at 90 days and CMBs in AIS patients who underwent reperfusion therapy. **E.** Sensitivity Analyses for Meta-Analyses on the Association between 90 day mortality CMBs in AIS patients who underwent reperfusion therapy. Abbreviations: CMB = cerebral microbleed, AIS = acute ischaemic stroke, IVT = intravenous thrombolysis, sICH = symptomatic intracerebral haemorrhage, HT = haemorrhagic transformation, CI = confidence interval.
